# Multicenter Evaluation of the Solana Group A Streptococcus Assay: Comparison with Culture

**DOI:** 10.1128/JCM.01268-16

**Published:** 2016-08-24

**Authors:** Timothy S. Uphoff, Blake W. Buchan, Nathan A. Ledeboer, Paul A. Granato, Judy A. Daly, Tara N. Marti

**Affiliations:** a Molecular Pathology, Marshfield Labs, Marshfield, Wisconsin, USA; b Pathology & Laboratory Medicine, Medical College of Wisconsin, Milwaukee, Wisconsin, USA; c Laboratory Alliance of Central New York, Liverpool, New York, USA; d Department of Pathology, University of Utah, Salt Lake City, Utah, USA; e Primary Children's Medical Center, Salt Lake City, Utah, USA; Memorial Sloan-Kettering Cancer Center

## Abstract

We compared group A Streptococcus (GAS) culture with a rapid helicase-dependent amplification (HDA) method using 1,082 throat swab specimens. The HDA method demonstrated 98.2% sensitivity and 97.2% specificity. GAS prevalence by culture was 20.7%, and it was 22.6% using the HDA method. In 35 min, the HDA method provided rapid, sensitive GAS detection, making culture confirmation unnecessary.

## TEXT

Group A Streptococcus (GAS) (Streptococcus pyogenes) is the most common bacterial cause of acute pharyngitis in school-aged children, affecting approximately 1 in 10 children per year (1, 2). Besides pain and discomfort, GAS pharyngitis can lead to suppurative complications, such as otitis media and peritonsillar abscess, and to nonsuppurative sequelae, such as rheumatic fever (3). Rapid, accurate detection of GAS is critical, since early treatment with appropriate antibiotics can reduce symptom severity and risk of complications (4–8). Additionally, accurate diagnosis can reduce unnecessary antibiotic use, as most cases of pharyngitis are viral (9, 10). Diagnosis of GAS pharyngitis using clinical signs alone is unreliable; physicians miss up to 50% of GAS pharyngitis cases and identify 20% to 40% of non-GAS sore throat cases as requiring antibiotics (11, 12). The standard procedure for laboratory detection of GAS, culture on blood agar, typically requires 24 to 48 h. Physicians, therefore, treat patients presumptively while awaiting culture results or withhold antibiotic therapy until GAS is confirmed with culture. Since the 1980s, commercial rapid antigen Streptococcus tests (RASTs) have been available for GAS detection. The advantage of these tests is that they can be quickly performed in the physician's office. While RASTs often have good specificity (>95%), they have a lower sensitivity (∼85%) than that of culture and, thus, require culture confirmation of negative tests (13–15). Recently, several manufacturers introduced molecular amplification methods for GAS detection (16–18). Herein, we evaluate another molecular GAS assay for rapid detection of GAS without the need for culture confirmation. The assay is performed using the Solana instrument, with which the GAS DNase B (*sdaB*) target gene sequence is amplified by an isothermal helicase-dependent amplification (HDA) reaction in the presence of an internal process control sequence.

We prospectively collected throat swab specimens submitted for GAS detection from symptomatic patients at four sites across the United States. Specimens were collected on polyester, nylon, or rayon swabs and transported to the laboratory in Amies, Stuarts, or ESwab transport medium. All samples were tested within 48 h of collection using the Solana GAS assay according to the manufacturer's instructions. The samples were also tested using standard GAS culture according to the *Clinical Microbiology Procedures Handbook* (19). Plates were incubated (35°C to 37°C, 5% CO_2_) and observed at 24 and 48 h. Colonies with an appearance typical of GAS were identified using Gram stain, catalase, and latex-typing tests. Residual transport medium was also sent to Quidel for culture of all specimens. A positive culture result found in either laboratory was sufficient to deem the sample culture positive. GAS PCR was performed on samples with discordant culture and HDA results. For these samples, a sterile swab was placed in the transport tube to saturate the swab, which was then tested using the Lyra direct strep assay according to the manufacturer's instructions.

The mean age of patients in this study was 15 years (range, <2 to 94 years), and 56% were female. Of the 1,082 samples tested, one gave invalid results even after repeat testing, indicating some type of amplification inhibition. Of the remaining specimens, 220 samples were positive by both culture and the Solana assay. Twenty-four samples were positive by the Solana assay but negative by culture, and four were negative by the Solana assay but positive by culture. When tested with the FDA-cleared Lyra GAS PCR assay, which has a genetic target different than that of the Solana GAS assay, 16 of 24 Solana-positive/culture-negative samples were positive for GAS. It should be noted that three of the four culture-positive/Solana-negative samples were not confirmed by Lyra PCR analysis. Negative Lyra PCR results may have been due to freezing of the sample, lack of residual sample, or target differences between the two molecular assays. A limitation of this study is that PCR was not performed on all samples, only those with discordant culture and Solana methods results. Compared with culture, the Solana HDA method overall demonstrated 98.2% (95% confidence interval [CI, 95.5% to 99.3%]) sensitivity and 97.2% (95% CI, 95.9% to 98.1%) specificity (see Table 1), as calculated using the exact test method (see Table 1). The HDA method generated a higher positivity rate than culture (22.6% versus 20.7%). When analyzed by transport medium type, the results were very similar. After discordant adjudication, the HDA method demonstrated 100% sensitivity and 97.9% specificity compared with culture using samples collected in Amies transport medium (data not shown). For samples collected in ESwab and Stuarts media, the adjudicated sensitivities were 97.9% and 100% and specificities were 98.8% and 99.6%, respectively. Solana HDA precision testing was performed using a four-member panel containing negative, high-negative (0.3× limit of detection [LoD]), low-positive (1× published LoD [6.81 × 10^4^ CFU/ml], determined elsewhere), and moderate-positive (3× LoD) samples along with positive and negative controls. This panel was tested in triplicate by two operators on five consecutive days at three independent sites (540 determinations). Precision studies demonstrated excellent reproducibility and no failures. All samples tested at or above the established LoD gave positive results, while 64% of the high-negative samples (0.3× LoD) were positive (see Table 2).

**TABLE 1:** Performance of all samples in the Solana GAS assay compared with culture

Solana GAS assay	Composite culture		
	No. positive^*a*^	No. negative^*b*^	Total No.
Positive	220	24^*c*^	244
Negative	4^*d*^	833	837
Total	224	857	1,081

a Sensitivity, 220/224 (98.2% [95% CI, 95.5% to 99.3%]).

b Specificity, 833/857 (97.2% [95% CI, 95.9% to 98.1%]).

c Of the 24 discordant specimens, 16 were positive for GAS when tested with an additional FDA-cleared molecular device; 8 were negative.

d Of the 4 discordant specimens, 3 were negative when tested with an additional FDA-cleared molecular device.

**TABLE 2:** Precision results of Solana GAS assay

Category	Site 1		Site 2		Site 3		Overall		95% CI
	No. positive/No. tested	% positive	No. positive/No. tested	% positive	No. positive/No. tested	% positive	No. positive/No. tested	% positive	
High negative (0.3× LoD)	24/30	80	20/30	67	14/30	47	58/90	64	54–74
Low positive (LoD)	30/30	100	30/30	100	30/30	100	90/90	100	96–100
Moderately positive (3× LoD)	30/30	100	30/30	100	30/30	100	90/90	100	96–100
Negative	0/30	0	0/30	0	0/30	0	0/90	0	0–4
Positive control	30/30	100	30/30	100	30/30	100	90/90	100	96–100
Negative control	0/30	0	0/30	0	0/30	0	0/90	0	0–4

Current RASTs demonstrate insufficient sensitivity for ruling out GAS infections without culture confirmation. Thus, the speed and simplicity of RASTs are not achieved for the vast majority of patients. Traditional PCR techniques match the sensitivity of culture but require costly thermocycling equipment and often specialized training. The Solana GAS assay demonstrated excellent sensitivity and reproducibility compared to those of traditional bacterial culture and matched the speed of PCR. The simplicity of this method and the fact that it does not require costly thermocycling instrumentation should make it more broadly accessible to relatively small laboratories than traditional PCR is currently. Implementing a method such as the Solana GAS assay can reduce turnaround times, delays in effective therapy, and unnecessary antibiotic use. Additional studies comparing this with other available rapid molecular methods for GAS detection, such as those used with the *illumi*gene or Simplexa platform, would be of interest.
